# Exploring the causal role of intimate partner violence and abuse on depressive symptoms in young adults: a population-based cohort study

**DOI:** 10.1186/s12916-021-02182-3

**Published:** 2022-01-10

**Authors:** Annie Herbert, Jon Heron, Maria Barnes, Christine Barter, Gene Feder, Khadija Meghrawi, Eszter Szilassy, Abigail Fraser, Laura D. Howe

**Affiliations:** 1 Department of Population Health Sciences, University of Bristol, Bristol, UK; 2 MRC Integrative Epidemiology Unit at the University of Bristol, Oakfield House, Oakfield Grove, Bristol, BS8 2BN UK; 3 Centre for Academic Primary Care, University of Bristol, Bristol, UK; 4 Connect Centre, University of Central Lancashire, Preston, UK; 5 Bristol Medical School, University of Bristol, Bristol, UK; 6 NIHR Biomedical Research Centre, University of Bristol, Bristol, UK

**Keywords:** Intimate partner violence, Young adult, Cohort studies, Depressive disorder

## Abstract

**Background:**

Previous studies have shown an association between experience of intimate partner violence and abuse (IPVA) and depression. Whether this is a causal relationship or explained by prior vulnerability that influences the risk of both IPVA and depression is not known.

**Methods:**

We analysed data from the Avon Longitudinal Study of Parents and Children prospective cohort (*N* = 1764 women, 1028 men). To assess the causal association between IPVA at 18–21 years old and logged depressive symptom scores at age 23, we used (i) multivariable linear regression, (ii) inverse probability of treatment weighting (IPTW), and (iii) difference-in-difference (DiD) analysis, which compared the mean change in logged depressive symptom scores between ages 16 and 23 between those who experienced IPVA and those who did not.

**Results:**

Women who experienced IPVA had on average 26% higher depressive symptom scores after adjustment for measured confounders (ratio of geometric means 1.26, *95% CI* 1.13 to 1.40). In men, the difference was 5% (ratio of geometric means 1.05, *95% CI* 0.92 to 1.21). Results from IPTW analysis were similar. In the DiD analysis, there was no evidence that being exposed to IPVA affected the change in depressive symptom scores over time compared to being in the non-exposed group for either women (difference-in-differences 1%, −12 to 16%) or men (−1%, −19 to 20%).

**Conclusions:**

Multivariable linear regression and IPTW suggested an association between IPVA and higher depressive symptom score in women but not men, but DiD analysis indicated a null effect in both women and men. This suggests the causal origins of higher depressive symptoms in this young adult population are likely to reflect prior vulnerability that leads to both higher depressive symptoms and increased risk of IPVA exposure.

**Supplementary Information:**

The online version contains supplementary material available at 10.1186/s12916-021-02182-3.

## Background

Intimate partner violence and abuse (IPVA) is experienced by approximately 30% of women and 24% of men between 18 and 21 years of age in the UK [1]. IPVA may have lasting consequences for mental health, yet there is a lack of long-term studies on the effect of IPVA on depressive symptoms. Depression is the most prevalent mental health problem worldwide [2] and is experienced by 22.5% of women and 16.8% of men in the UK [3]. Although longitudinal studies investigating the relationship between IPVA and depression consistently report bidirectional associations, they usually do not have strong study designs and robust analytical approaches for assessing causality [4]. Confounding may influence these findings if background factors such as adversity in childhood influence both the risks of experiencing IPVA and depressive symptoms.

Some longitudinal evidence is available from younger adolescent samples [5], suggesting an increase in depressive symptoms following IPVA, at 5 years following IPVA, after adjusting for baseline depression [6, 7]. One study that additionally adjusted for other potentially confounding factors (race, age, socioeconomic status, childhood maltreatment, and pubertal status) reported small increases in mean Centre for Epidemiology Studies Depression scores at 5 years following psychological or physical IPVA [7]. However, the number and range of factors adjusted for in this analysis may not fully capture the large range of factors that can influence both IPVA and depression. Therefore, concerns remain about the extent to which associations between IPVA and depressive symptoms in young adults are causal. This is important to establish because it has implications for interventions. If associations are not causal, whilst prevention of IPVA and support to survivors is still essential, we also need effective interventions in earlier stages of the life course to prevent adult mental health problems.

Here, we use data from a UK general population-based birth cohort to investigate the longitudinal relationship between IPVA at ages 18–21 and depressive symptoms at age 23, using three techniques—multivariable linear regression, inverse probability of treatment weighting, and a difference-in-difference model. Women are more likely than men to experience IPVA [1, 8] and to have higher levels of depressive symptoms [9]. Furthermore, women who experience IPVA are more likely to report that it had a negative impact on them than men who experience IPVA [1]. Therefore, we conduct all analyses separately for women and men.

## Methods

### Data and participants

We analysed data from the Avon Longitudinal Study of Parents and Children (ALSPAC) cohort. Around 14,500 pregnant women residing in Avon, UK, with expected delivery dates in April 1991–December 1992 (approximately three-quarters of the eligible population) were recruited. When the oldest children were approximately 7 years of age, an attempt was made to bolster the initial sample with eligible cases who had failed to join the study originally, resulting in an additional 913 children being enrolled. Information has been regularly collected since enrolment until the present. Study data were collected and managed using REDCap electronic data capture tools hosted at the University of Bristol [10]. More information on both the mothers and their offspring is available in published cohort profiles [11–13]. The study website contains details of all the data that are available through a fully searchable data dictionary and variable search tool [14].

Within questionnaires offered in both online and paper format, 3280 ALSPAC participants answered questions on IPVA at age 21 (interquartile range for age of response 21 to 22). These questions ask about IPVA both prior to turning 18 and between ages 18 and 21. In order to make causal inferences, it was important to establish the timing of exposure to IPVA such that we could account for confounding; therefore, we excluded participants exposed to IPVA prior to turning 18 (*n* = 487) after excluding one participant whose sex could not be determined, this left a final analysis sample of 2792 participants.

### Exposure: IPVA

We use the term ‘IPVA’ to mean psychological, physical, and/or sexual abuse carried out by an intimate (romantic) partner. At age 21, ALSPAC participants were asked about IPVA. For example, how often an intimate partner had ‘Told you who you could see and where you could go and/or regularly checked what you were doing and where you were (by phone or text)?’, to which one could respond ‘never’, ‘once’, ‘a few times’, or ‘often’, and whether this occurred prior to turning 18, after turning 18, or at both time points. These questions have been previously developed based on previous UK and European questionnaires and the PROVIDE questionnaire [15, 16] and are described in full in Additional file 1: Box S1, as well as a report of their psychometric properties [17]. The questions are provided in Additional file 1: Box S1. As in previous work, we considered any response of at least ‘once’, to any of the eight questions as exposure to IPVA, because the header of the questionnaire was ‘Intimate Partner Violence’, likely raising the threshold of severity for reporting certain behaviours, and because for participants who answered at least ‘once’ to any of the questions, a negative impact was reported by 75–99% [1].

We also included different IPVA types (psychological, physical, sexual) in analyses. The questions used to distinguish different types have been described previously [17]. Combinations of types were then grouped based on both the sample prevalence of different combinations and existing literature finding variation in impact and mental health between such combinations [18, 19]. The categories of IPVA types analysed were no victimisation, psychological victimisation only, physical victimisation (whether with or without psychological victimisation, but with no sexual victimisation), and any sexual victimisation.

### Outcome: depressive symptom scores

Depressive symptoms were captured at age 23 via Moods and Feelings Questionnaire (MFQ) scores [20, 21]. This questionnaire asks 13 questions about depressive symptoms in the past 2 weeks, with response options ‘not true’, ‘somewhat true’, or ‘true’ (scoring 0, 1, and 2, respectively).

### Other variables

Covariates were included in models either to estimate adjusted coefficients or to create propensity scores (described later under ‘Inverse probability of treatment (IPTW)’).

These covariates were known risk factors for IPVA and depressive symptoms (i.e. could confound the relationship between the two) based on the previous empirical literature, or, as recommended by the propensity score methods literature [22, 23], variables that were known risk factors for, as a minimum, the outcome (i.e. depressive symptoms) [24–28]. These were all coded as binary variables: socioeconomic status at birth [Index Multiple Deprivation quintiles 4–5 vs. 1–3; based on the mother’s postcode at the time of the child's birth], ethnicity [White vs. Person of Colour; self-reported by the mother during pregnancy], sexual minority [100% heterosexual vs. others; self-reported by the young person in a questionnaire at age 15], anxiety (Clinical Interview Schedule-Revised; administered via a self-reported computer questionnaire at a research clinic at age 17; defined as any of the following anxiety disorders: generalised anxiety disorder, social phobia, specific (isolated) phobia, panic disorder, or agoraphobia, according to the International Classification of Diseases, 10th revision criteria), extreme parental monitoring at age 15 (Parental Monitoring Questionnaire score; > 13 considered extreme [29]), anti-social behaviour at age 14 (defined as self-reported perpetration of one or more of a list of anti-social behaviours (see Additional file 1: Box S2) at least two times in the past 12 months), smoking at age 16 [self-reported; at least weekly vs. less than weekly], cannabis use at age 16 [self-reported; at least weekly vs. less than weekly], illicit (non-cannabis) drug use at age 16 [self-reported; any past month vs. none in past month], hazardous alcohol use at age 18 (self-reported Alcohol Use Disorders Identification Test score; ≥8), adverse childhood experiences (ACEs) at ages 0–16 [ten different variables: emotional abuse, physical abuse, sexual abuse, emotional neglect, bullying, witnessing domestic violence, parental mental health problem, parental substance abuse, parental criminal conviction, and parental separation; these variables are based on multiple prospectively and retrospectively reported questionnaires; see references for further details] [24, 26–28], low self-esteem at age 17 (Bachman Self Esteem Scale score ≤29) [30], overweight at age 17 (body mass index ≥ 25 kg/m^2^, assessed at a research clinic), sleep problems at age 17 (Clinical Interview Schedule-Revised sleep subsection score ≥2) [31, 32], and parents’ education level (at least one parent with at least O level qualifications; reported by mother and her partner during pregnancy) [17]. We also included a measure of depressive symptoms prior to exposure (MFQ score at age 16, as having prior depressive symptoms is a risk factor for IPVA and later depression). More detail on how the above variables were derived using ALSPAC data has been published previously [1, 33]. Distributions and levels of missing data for each of these covariables are presented in Additional file 1: Table S1 [1, 20, 21, 30–32, 34].

### Statistical analysis methods

We carried out all analyses separately for women and men, given that some outcomes of IPVA have been shown to differ by sex, e.g. reported impact of IPVA [1]. As per disclosure rules for use of ALSPAC data, we do not report any numbers (or related percentages) less than 5. R scripts used in these analyses are available at https://github.com/pachucasunrise/IPVA_depression.

We employed the following three methods to test the causality of the effect of IPVA on depression.

1. *Multivariable linear regression*. We fitted a model where depressive symptom score at age 23 was the dependent variable and IPVA at ages 18–21 was the independent variable. An unadjusted model was compared with two adjusted models: ‘Adjusted Model A’ included as covariates that had been adjusted for within existing literature [6, 7]: MFQ score at age 16, ethnicity, socioeconomic status, and four ACEs representing child maltreatment (emotional abuse, physical abuse, sexual abuse, emotional neglect). Adjusted model B included all covariates listed in the ‘Other variables’ section.
2. *Inverse probability of treatment weighting (IPTW)*. A linear regression was fitted where the outcome was depression score at age 23 and (IPVA at 18–21 vs. none) was included as an independent variable. Participants were weighted using stabilised weights calculated as a function of the propensity to experience IPVA (estimated from a separate logistic regression model that included all relevant measured covariates as independent variables) and the global probability of IPVA [35]. Further information on IPTW and the methods used are in Additional file 1: Box S3 [22, 35–37].
3. *Difference-in-differences (DiD)*. Here, we fitted a linear regression model where each individual contributed two data points on the ‘outcome’ (depression score), one at 16 years, before the time period for exposure, and one after at age 23 [34–36]. The assumption is that the difference between groups (IPVA at ages 18–21 vs. none) in depressive symptom scores at baseline (prior to exposure) represents all confounding, both measured and unmeasured. The difference in the change in depression scores between 16 and 23 years between those exposed to IPVA and those not exposed (difference-in-difference', estimated as an interaction between the exposure and time) is then assumed to reflect the causal effect of IPVA on change in depression score, under a set of assumptions. Further information on DiD and the methods used are described in Additional file 1: Box S4 [38–40].

We repeated the above three methods for categories of young adult IPVA victimisation types. For IPTW, we used multinomial logistic regression to estimate propensity scores for different categories. For DiD analysis, we fitted three difference models for each of the categories of IPVA victimisation types vs. no IPVA victimisation.

We use the natural log of depressive symptom scores due to its non-normal distribution. The exponentiated regression coefficient for a logged outcome represents the ratio of geometric means. Using logged depressive symptom scores, residual histograms were adequately normally distributed and *p* values from the Kolmogorov-Smirnoff test for the fully adjusted model (multivariable linear regression adjusted model B) were 0.90 and 0.72 for women and men, respectively.

To facilitate comparison with the existing literature, we present the odds ratio for the association of IPVA with a dichotomised measure of depression (defined as depressive symptom score above 12 [41]).

### Missing data

As the data used for this study are collected over a 23-year period, there is inevitably loss to follow-up and missing data. Participants were included in analyses if they had data on depressive symptom scores at ages 16 and 23, IPVA exposure, and ethnicity. For other covariates, we imputed any missing values using multiple imputation via chained equations, separately for women and men. We imputed 50 datasets using the MICE package in R [42], with 10 iterations. Regression models were fitted in each of these imputed datasets and coefficients and standard errors pooled using Rubin’s rules [43]. Where *p* values were reported, this was the median *p* value between the 50 imputed datasets.

For IPTW, within each imputed dataset, propensity scores and stabilised weights were estimated and regressions inverse-weighted, before pooling resulting coefficients across the imputed datasets [44].

### Sensitivity analyses

We repeated the main analysis within completely observed data and again using the non-transformed variable of depressive symptom scores.

The main analysis was restricted to those who reported no IPVA victimisation before age 18. People exposed to IPVA both before age 18 and at ages 18–21 may differ in other ways to those who were only exposed at ages 18–21. We repeated analyses without this restriction (*N* = 3279 vs. 2792). However, it must be noted that we can now no longer assume that the variables adjusted for or the difference in mean depression score at age 16 occurred prior to the IPVA exposure. Therefore, we cannot make causal inference from those results.

Of the participants included in our main analysis, 90% reported that they had had at least one intimate relationship/encounter by age 21 (*N* = 2421). We include all participants irrespective of their reported relationships in the main analysis because the information on relationships is derived from multiple questionnaire items, most of which were not directly intended to assess whether a participant had experienced an intimate relationship. We therefore believe it is likely that our indicator variable misses many young people who have been in an intimate relationship. Sensitivity analyses that restricted to the 2421 participants who were sure have had at least one intimate relationship are presented to more robustly meet the positivity assumption of causality (i.e. that all individuals in the sample can be exposed to IPVA).

To assess the common trend assumption for DiD analysis, we checked that differences in mean logged depressive symptom scores were similar between the ages 13 and 16 for people who did and did not report IPVA victimisation at 18–21 years.

To assess whether results differed according to the severity of IPVA experienced, we repeated our analysis categorising people according to whether they reported 0, 1, 2, or 3 types of IPVA. To examine whether the associations between IPVA and depressive symptoms differed according to previous experience of maltreatment, we repeated our analysis stratified according to a binary indicator of child maltreatment (defined as emotional abuse, physical abuse, sexual abuse, or emotional neglect by anyone other than an intimate/romantic encounter by the age of 16 [1, 33]). These two sensitivity analyses were performed only in people with complete data on all variables used in analyses because small numbers in some strata wouldn't allow for the appropriate imputation models (e.g. including relevant interaction terms).

## Results

### Cohort characteristics

Of 1764 women and 1028 men in the study cohort, 482 (27%) and 210 (20%) reported that they had been victimised between ages 18 and 21, respectively; characteristics of participants according to whether or not they experienced IPVA victimisation are presented in Additional file 1: Table S1 [20, 21, 30–32, 34, 45].

### Relationship between victimisation and subsequent depression scores/binary measure of depression

The geometric mean depressive symptom scores at age 23 were 5.2 for both women and men in the non-victimised group and 7.0 and 6.2 in women and men who experienced IPVA victimisation. In women, IPVA was associated with a doubling in the odds of depressive symptoms above the threshold for defining depression (*OR* 2.10, *95% CI* 1.57 to 2.81). In men, IPVA was associated with a 36% increase in the odds of depression (*OR* 1.36, *95% CI* 0.91 to 2.04) (Table 1).

**Table 1:** Statistics on depressive symptom scores and a binary depression measure at age 23, stratified by sex and reporting of IPVA victimisation at ages 18–21

	***N***	Continuous depressive symptoms					Binary measure of depression^**a**^
		Median	***IQR***	Arithmetic mean	Standard deviation	Geometric mean	***OR*** (***95% CI***)
**Women**							
No IPVA	1329	4.96	(2.00 to 8.80)	6.13	5.51	5.17	2.10 (1.57 to 2.81)
IPVA	435	6.28	(3.10 to 12.19)	8.39	6.52	7.01	
**Men**							
No IPVA	771	4.70	(2.00 to 9.82)	6.41	5.91	5.19	1.36 (0.91 to 2.04)
IPVA	435	5.62	(3.00 to 11.00)	7.44	6.05	6.21	

Statistics are pooled from 50 multiply imputed datasets using Rubin’s rules

*CI* Confidence interval, *IPVA* Intimate partner violence and abuse, *IQR* Interquartile range, *OR* Odds ratio

^a^Depression is defined as a score of 13 or more on the Short Mood and Feelings Questionnaire

#### Multivariable linear regression

Depressive symptoms at age 23 were 36% higher in women and 20% higher in men who reported being victimised at ages 18–21 compared to those who had not reported being victimised (ratio of geometric means in women 1.36, *95% CI* 1.23 to 1.51; men 1.20, 1.04 to 1.37) (Table 2, crude models). The estimated difference in depressive symptom scores between victimised and non-victimised groups was reduced after adjusting for logged depressive symptom scores at age 16, ethnicity, socioeconomic status, and certain ACEs; the association still remained in women (26%; ratio of geometric means 1.26, *95% CI* 1.13 to 1.40), but was attenuated to the null in men (6%; ratio of geometric means 1.06, *95% CI* 0.92 to 1.21) (Table 2, adjusted model A); these results remained relatively unchanged after adjusting for the larger set of covariates in adjusted model B.

**Table 2:** Association between IPVA victimisation at ages 18–21 and logged depressive symptom score at age 23 using linear regression and IPTW. Analysis on multiply imputed data, *N* = 1764 women and 1028 men

	Women		Men	
Model	% change	Ratio of geometric means (***95% CI***)	% change	Ratio of geometric means (***95% CI***)
1 (crude)	36	1.36 (1.23, 1.51)	20	1.20 (1.04, 1.37)
2 (adjusted A^a^)	26	1.26 (1.13, 1.40)	6	1.06 (0.92, 1.21)
3 (adjusted B^b^)	26	1.26 (1.13, 1.40)	5	1.05 (0.92, 1.21)
4 (IPTW)	20	1.20 (1.01, 1.43)	5	1.05 (0.84, 1.32)

Depressive symptom score was logged, and the coefficients from linear regression models were exponentiated to ratios of geometric means and subsequently converted to percent changes in the geometric mean of depressive symptom score; % change = [exp(coefficient)−1] × 100. Statistics are pooled from 50 multiply imputed datasets using Rubin’s rules, as described in the ‘Methods’ section

*IPTW* inverse probability of treatment weighting

^a^Adjusted for logged depressive symptom score at age 16, ethnicity, socioeconomic status, and dummy variables for childhood emotional abuse, physical abuse, sexual abuse, and emotional neglect

^b^As in adjusted A* and additionally adjusted for sexual minority status, anxiety, extreme parental monitoring, anti-social behaviour, smoking, cannabis use, illicit (non-cannabis) drug use, hazardous alcohol use, bullying, witnessing domestic violence, parental mental health problem, parental substance abuse, parental criminal conviction, and parental separation

#### IPTW

When regression estimates were inverse probability of treatment weighted, associations between IPVA and depressive symptoms were in line with the multivariable-adjusted results (women 20%; ratio of geometric means 1.20, *95% CI* 1.01 to 1.43; men 5%, ratio of geometric means 1.05, *95% CI* 0.84 to 1.32).

#### DiD

**Fig. 1:**
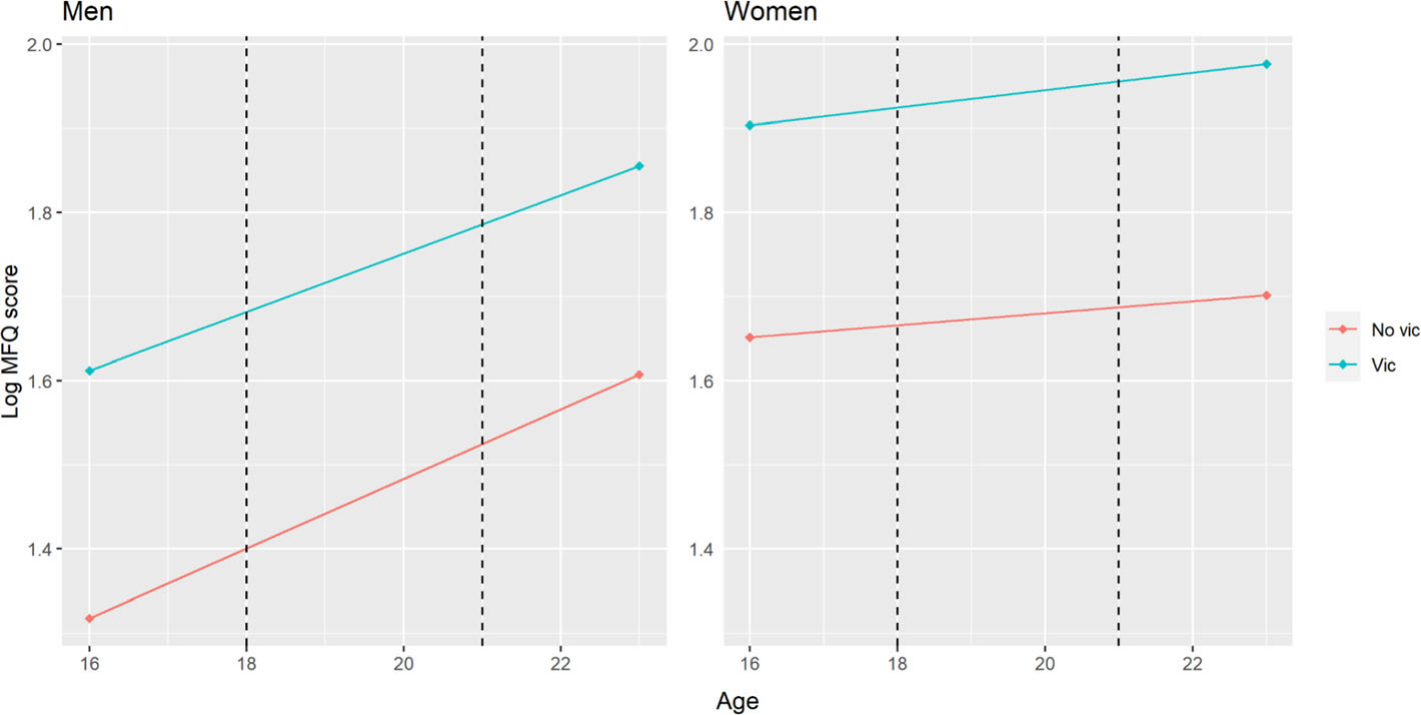
Estimates from difference-in-difference analysis for the relationship between IPVA victimisation at ages 18–21 and logged depressive symptom scores at ages 16 and 23. Dashed lines represent ages 18–21 (when the exposure IPVA was reported to occur). The parallel nature of the lines suggests that IPVA between ages 18 and 21 years does not have a causal effect on increasing depressive symptoms. Evidence of a positive causal effect would be provided by the lines for people who experienced IPVA (‘vic’) and those who did not experience IPVA (‘No vic’) fanning in or out over time

Depressive symptoms prior to IPVA victimisation (age 16) were 29% higher in women who went on to experience IPVA victimisation between 18 and 21 years (ratio of geometric means 1.29, *95% CI* 1.17 to 1.42) and 34% higher in men who went on to experience IPVA victimisation between 18 and 21 years (ratio of geometric means 1.34, *95% CI* 1.16 to 1.55) (Table 3; Fig. 1). Depressive symptom scores increased over time in men, but less so in women. There was little evidence of a difference in differences (i.e. that being in the victimised group affected the change in geometric mean depressive symptom scores over time compared to being in the non-victimised group; the interaction between victimisation and time), for either women (2%, ratio of geometric means 1.02, *95% CI* 0.89 to 1.18, *p* value = 0.76) or men (−5%, ratio of geometric means 0.95, *95% CI* 0.77 to 1.18, *p* value = 0.67).

**Table 3:** Difference-in-difference analysis for the relationship between IPVA victimisation at ages 18–21 and logged depressive symptom scores at ages 16 and 23 (models fitted in imputed datasets, *N* = 1764 women and 1028 men)

	Women			Men		
Term	% change^**Ϯ**^	Ratio of geometric means (***95% CI***)	***p*** value	% change^**Ϯ**^	Ratio of geometric means (***95% CI***)	***p*** value
IPVA^¥^	29	1.29 (1.17, 1.42)	< 0.001	34	1.34 (1.16, 1.55)	< 0.001
Time^¥¥^	5	1.05 (0.98, 1.13)	0.18	34	1.34 (1.22, 1.47)	< 0.001
IPVA*Time^¥¥¥^	2	1.02 (0.89, 1.18)	0.76	−5	0.95 (0.77, 1.18)	0.665

*CI* confidence interval

*An interaction between two variables

^Ϯ^In geometric mean. % change calculated from the estimated coefficient for victimisation in each model, as % change = [exp(coefficient) − 1] × 100

^¥^The difference in depressive symptoms at baseline (age 16 years) comparing people exposed to IPVA with people not exposed to IPVA

^¥¥^The change in depressive symptoms over time, in people not exposed to IPVA

^¥¥¥^The difference in difference, i.e. the difference in the change in depressive symptoms over time, comparing people exposed to IPVA with people not exposed to IPVA. This is estimated as an interaction between IPVA and time

### Relationship between different categories of victimisation types and subsequent depression scores

Of 1764 women and 1028 men in the study cohort, 209 women (12%) and 123 men (12%) reported being psychologically victimised only at ages 18–21; 111 (6%) and 50 (5%) reported being physically victimised, with or without experiencing psychological victimisation but without sexual victimisation; and 162 (9%) and 37 (4%) reported being sexually victimised.

#### Multivariable linear regression

The geometric mean was 22% and 5% higher for women and men who were psychologically victimised only, 37% and 20% higher for women and men physically victimised (whether psychologically victimised or not, but not sexually victimised), and 55% and 47% higher for women and men who were sexually victimised (Table 4). These associations attenuated after adjustement, those for sexual victimisation roughly halved. Most associations were imprecisely estimated due to the small number of people experiencing each subtype of victimisation, particularly any sexual victimisation, and in men.

**Table 4:** Regression estimates for the relationship between different categories of IPVA types at ages 18–21 and logged depressive symptom score at age 23 (models fitted in imputed data, *N* = 1764 women and 1028 men)

		Women		Men	
Model	Subtype of IPVA	% change	Ratio of geometric means (***95% CI***)	% change	Ratio of geometric means (***95% CI***)
1 (crude)	Psych only	22	1.22 (1.06, 1.41)	5	1.05 (0.87, 1.28)
	Physl (with or without psych, no sex)	37	1.37 (1.13, 1.66)	20	1.20 (0.92, 1.57)
	Any sexual	55	1.55 (1.29, 1.87)	47	1.47 (1.17, 1.86)
2 (adjusted A*)	Psych only	25	1.25 (1.08, 1.44)	−4	0.96 (0.79, 1.16)
	Phys (with or without psych, no sex)	31	1.31 (1.08, 1.58)	12	1.12 (0.87, 1.43)
	Any sexual	22	1.22 (1.01, 1.48)	20	1.20 (0.96, 1.50)
3 (adjusted B**)	Psych only	28	1.28 (1.11, 1.48)	−4	0.96 (0.79, 1.16)
	Phys (with or without psych, no sex)	29	1.29 (1.06, 1.56)	10	1.10 (0.86, 1.41)
	Any sexual	18	1.18 (0.98, 1.43)	20	1.20 (0.96, 1.50)
4 (IPTW)	Psych only	19	1.19 (1.02, 1.39)	0	1.00 (0.81, 1.24)
	Phys (with or without psych, no sex)	11	1.11 (0.90, 1.38)	−3	0.97 (0.72, 1.30)
	Any sexual	1	1.01 (0.83, 1.23)	43	1.43 (1.13, 1.81)

Statistics are pooled from 50 multiply imputed datasets using Rubin’s rules, as described in the ‘Methods’ section. % change calculated from the estimated coefficient for IPVA victimisation category in each model, as % change = [exp(coefficient) − 1] × 100

*Any sexual* any sexual IPVA victimisation (with or without psychological or physical IPVA victimisation), *CI* confidence interval, *IPTW* inverse probability of treatment weighting, *Phys* physical IPVA victimisation (with or without psychological, no sexual), *Psych only* psychological victimisation only

*Adjusted for logged depressive symptom score at age 16, ethnicity, socioeconomic status, and dummy variables for childhood emotional abuse, physical abuse, sexual abuse, and emotional neglect

**As in adjusted A* and additionally adjusted for sexual minority status, anxiety, extreme parental monitoring, anti-social behaviour, smoking, cannabis use, illicit (non-cannabis) drug use, hazardous alcohol use, bullying, witnessing domestic violence, parental mental health problem, parental substance abuse, parental criminal conviction, and parental separation

#### IPTW

When regression estimates were inverse probability of treatment weighted, most estimates attenuated (Table 4). One exception was the estimate for sexual victimisation in men (which increased from a 20% higher geometric mean depressive symptom scores in adjusted model B to 43% in the IPTW model, *95% CI* for the ratio of geometric means 1.13 to 1.81).

#### DiD

There was no evidence that any victimisation subtype affected the change in depressive symptoms over time, for either women or men (Table 5).

**Table 5:** Difference-in-difference analyses for the relationships between different categories of IPVA victimisation types at ages 18–21 (vs. no victimisation) and logged depressive symptom scores at ages 16 and 23 (models fitted in imputed datasets)

Model	Term	% change^**Ϯ**^	95% ratio of geometric means (***95% CI***)	***p*** value
**Women**				
1 (*N* = 1480)	Psych (vs. no IPVA)^¥^	9	1.09 (0.95, 1.26)	0.204
	Time^¥¥^	5	1.05 (0.98, 1.13)	0.180
	Psych*Time^¥¥¥^	3	1.03 (0.84, 1.26)	0.768
2 (*N* = 1382)	Phys (vs. no IPVA)^¥^	38	1.38 (1.15, 1.65)	0.001
	Time^¥¥^	5	1.05 (0.98, 1.13)	0.181
	Phys*Time^¥¥¥^	4	1.04 (0.80, 1.34)	0.784
3 (*N* = 1433)	Sexual (vs. no IPVA)^¥^	52	1.52 (1.31, 1.76)	< 0.001
	Time^¥^	5	1.05 (0.98, 1.13)	0.178
	Sexual*Time^¥¥¥^	0	1.00 (0.81, 1.23)	0.985
**Men**				
1 (*N* = 952)	Psych (vs. no IPVA)^¥^	24	1.24 (1.03, 1.49)	0.020
	Time^¥^	34	1.34 (1.22, 1.47)	< 0.001
	Psych*Time^¥¥¥^	−2	0.98 (0.75, 1.28)	0.895
2 (*N* = 879)	Phys (vs. no IPVA)^¥^	28	1.28 (0.98, 1.68)	0.070
	Time^¥¥^	34	1.34 (1.22, 1.47)	< 0.001
	Phys*Time^¥¥¥^	−8	0.92 (0.63, 1.34)	0.676
3 (*N* = 866)	Sexual (vs. no IPVA)^¥^	84	1.84 (1.36, 2.50)	< 0.001
	Time^¥¥^	34	1.34 (1.22, 1.47)	< 0.001
	Sexual*Time^¥¥¥^	−9	0.91 (0.59, 1.41)	0.673

*CI* confidence interval, *Phys* physical IPVA victimisation (with or without psychological IPVA, no sexual IPVA), *Psych* psychological IPVA victimisation only, *Sexual* any sexual IPVA victimisation (with or without psychological or physical IPVA victimisation)

*An interaction between two variables

^Ϯ^In geometric mean. % change calculated from the estimated coefficient for victimisation in each model, as % change = [exp(coefficient) − 1] × 100

^¥^The difference in depressive symptoms at baseline (age 16 years) comparing people exposed to each IPVA subtype with people not exposed to that IPVA subtype

^¥^The change in depressive symptoms over time, in people not exposed to the IPVA subtype

^¥^The difference in difference, i.e. the difference in the change in depressive symptoms over time, comparing people exposed to the IPVA subtype with people not exposed to the IPVA subtype

### Sensitivity analyses

Analyses using the crude depressive symptom score at age 23 (not logged) followed a similar pattern to when the outcome was logged (Additional file 1: Tables S2 and S3).

Results were similar when analyses were restricted to participants with complete/observed data on all variables used in analyses (‘complete case’) or when participants who reported no intimate relationships before age 21 were excluded (Additional file 1: Tables S4 and S5). When we included participants who had reported victimisation before age 18 (an exclusion criterion of the main analysis), IPTW models indicated that IPVA was associated with 43% higher depressive symptoms (ratio of geometric means 1.43, *95% CI* 1.29 to 1.58) amongst women and 26% (ratio of geometric means 1.26, *95% CI* 1.10 to 1.44) amongst men. However, the estimate from DID analysis including participants who had reported victimisation before age 18 was similar to the main analysis, suggesting that IPVA exposure was not associated with a different rate of change in depressive symptoms between 16 and 23 years.

There was no evidence to suggest a violation of the common trends assumption; *p* values for interaction between IPVA victimisation status and changes in depressive symptom score between 13 and 16 years were 0.33 for women and 0.59 for men (Additional file 1: Box S2).

Whilst unadjusted regression analyses suggested that depressive symptoms increased with greater number of types of IPVA experienced, the differences between experiencing three and experiencing one or two forms of IPVA reduced with adjustment for measured confounders (Additional file 1: Table S6), particularly for women. Furthermore, the difference-in-difference analysis did not demonstrate an increase in the change in depressive symptoms over time for women or men who experienced one, two, or three types of IPVA compared with those who did not experience IPVA (Additional file 1: Table S7 and Fig. S3). Sample sizes for these analyses were small; 54 women and 13 men reported all three types of IPVA.

In women, there as some evidence that there was an association between IPVA and depressive symptoms in people who had not experienced child maltreatment (Additional file 1: Table S8) but this was not supported by DiD analysis (Additional file 1: Table S9).

The association between IPVA and depressive symptoms was similar in women who had and had not experienced child maltreatment (Additional file 1: Table S8). In men, there was weak evidence that the association between IPVA and depressive symptoms was positive in people who had experienced child maltreatment, and negative in people who had not experienced child maltreatment (Additional file 1: Table S8) and that IPVA was associated with a faster rate of increase in depressive symptoms over time in those who had experienced child maltreatment (Additional file 1: Table S9). However, although numbers of men in these analyses were small and the confidence intervals were very wide.

## Discussion

Average depression scores at age 23 were 1–2 points higher for women and men who reported being victimised at ages 18–21 compared to those who did not amongst both women and men in a UK population-based birth cohort. When controlling for measured confounding using either multivariable linear regression or IPTW, analyses indicated a small association of IPVA victimisation with depressive symptom scores in women but not men. In contrast, DiD analyses, which can account for unmeasured time-fixed confounding, suggested no causal effect in either women or men. On balance, although women and men victimised at ages 18–21 were more vulnerable to depression at age 23, our data suggest that the associations observed in multivariable regression and IPTW analyses are likely affected by mismeasured and/or unmeasured confounders, and it is likely that the causal component of this relationship is, on average in this population, either small or null, and mainly driven by prior vulnerability that increased propensity to be exposed to IPVA. Sensitivity analyses suggested that the assumptions of the DID analysis were met and that our pattern of results remained the same in women who experienced multiple forms of IPVA and who had experienced child maltreatment in addition to IPVA. There was some suggestion that IPVA might be associated with a faster rate of change in depressive symptoms between ages 16 and 23 years in men who had previously experienced child maltreatment, but the number of men included in this analysis was small and associations were imprecisely estimated. Importantly, even if the null effect of the DiD analyses in our main analysis was driven by violated assumptions, the maximum possible causal effect for this population suggested by regression analyses would be around 0.9 points on the MFQ scale, a small effect, given a standard deviation of 5 to 6 points. This effect is much less than an assumed minimum important clinical difference of 5 points on the MFQ scale [46].

### Strengths and limitations

We analysed data on a large population-based cohort, using validated measures of IPVA and depression. A strength of this cohort is the availability of a range of different risk factors for IPVA and depression, and of depression itself, at multiple time points. This allowed us to carry out both IPTW and DiD, which has not been possible in previous datasets. Internal checks found no evidence of violation of the common trends assumption for DID analysis, but if this assumption was violated, we would anticipate it would likely bias the effect away from the null.

Depression scores were captured at age 23, 2–5 years following measured exposure to IPVA (depending on whether the participant was closer to age 18 or 21 at the time of exposure). It is possible that some participants did not develop depressive symptoms related to their IPVA exposure until after age 23. Similarly, as the IPVA may have occurred several years prior to measurement of depressive symptoms, some participants may have had recovering mental health by this time. A review of the published literature on trajectories of psychopathology following potentially traumatic events such as child maltreatment, cancer diagnosis, heart attack, or bereavement, indicated that an average of 65.7% of participants across studies experienced a ‘resilience’ trajectory, 20.8% experienced recovery, 10.6% experienced chronic symptoms, and 8.9% experienced delayed onset of symptoms [47].

Our findings may not generalise to older adults, where the relationship duration, living arrangements, financial responsibilities, and presence of children may differ. At the age at which depressive symptoms were captured in this study (23 years), depressive symptoms are far higher in women than in men and are on average stable or declining following a steep rise in adolescence [9].

Although we attempted to adjust for a wide range of confounding factors, other factors, for example traumatising experiences apart from the measured ACEs, may have confounded the multivariable regression and IPTW analyses. This is likely to underlie the differences between these analyses and the difference-in-difference analysis, the latter of which accounts for unmeasured confounding and, in general, suggests in our data that, on average in this population, there is limited evidence of a causal effect of IPVA on the change in depressive symptoms between ages 16 and 23 years.

There is a possibility that selection bias may have distorted effect estimates. Around one-third of individuals still in the cohort at 21 years old responded to the age 21 questionnaire; 77% of these participants had also responded to the age 16 questionnaire (which captured depression measures used in the DiD analyses). Given previous methodological work on the ALSPAC cohort indicating that those experiencing poorer socioeconomic and health outcomes will be less likely to participate [48], we could expect those with the worst depressive states and IPVA experiences would be less likely to participate. Indeed, individuals who did not respond to the age 21 questionnaire tended to have higher depressive symptom scores at age 16 than those who had responded; it was not possible to check this phenomenon for IPVA as it was not measured prior to age 21. If this were the case, and those with the worst IPVA experiences were less likely to participate, this would attenuate the effect of IPVA on depression, and so the ‘true’ general young adult population effect may in fact be larger. Thus, our null result may not generalise to a higher risk population. However, our sensitivity analyses demonstrated that, albeit in a small sample size with limited statistical power, there was no evidence of a causal effect of IPVA on change in depressive symptoms over time in higher risk women (women who experienced multiple forms of IPVA, or who experienced both child maltreatment and IPVA). There was, however, some suggestion that IPVA may result in increasing depressive symptoms in men who have previously experienced child maltreatment, but this finding requires replication in larger sample sizes. Despite being relatively affluent on average, the cohort still includes individuals with high depressive symptom scores (10% had scores of 10–12) or severe IPVA experiences [19, 21].

The retrospective nature of IPVA data may have resulted in some measurement error, including misclassification of whether IPVA occurred before or after age 18. We do not have data on service use or treatment for mental health in this cohort, nor on whether participants have ended the abusive relationships, and so we are unable to evaluate the degree to which this may explain the null relationship between IPVA and depressive symptoms.

### Comparison with literature

A systematic review and meta-analysis of longitudinal studies of the relationship between intimate partner violence and depression identified 16 studies with 36,163 participants [4]. Most of those studies were not restricted to young adults. All but one study identified a positive association between intimate partner violence and incident depressive symptoms in women, with a pooled odds ratio of 1.97 (*95% CI* 1.56–2.48). There was also evidence of an association between intimate partner violence and depression in men, but only two studies included data for men so meta-analysis was not performed. Our main analysis used a continuous measure of depressive symptoms, which is not directly comparable to the binary measures used in this meta-analysis, but when we dichotomised our measure of depressive symptoms, the odds ratio for the relationship between IPVA and depression was similar to the pooled result from the meta-analysis for both women and men, supporting the notion that our findings are not influenced by selection bias. In line with our conclusion about prior vulnerability influencing the association between IPVA and depressive symptoms, the meta-analysis also found evidence of an association between depressive symptoms and incident IPV (pooled odds ratio from four studies 1.93, *95% CI* 1.51–2.48).

Our findings are comparable with one of the only longitudinal studies we could identify that studied depression following IPVA in young people [7]. In the National Longitudinal Survey of Adolescent Health which surveyed a younger US population (aged 12–18) about psychological and physical IPVA, depression was captured using a different measure but with similarly worded questions and was on a similar scale [49, 50], and with similar follow-up. The authors found a difference of up to 0.9 points (depending on IPVA subtype, *95% CI*s ranging from 0.01 to 1.76) after adjustment for confounders equivalent to those we adjusted for in adjusted model A [7]. In this study, the associations of IPVA with depression were similar for women and men, although gender differences were seen for other outcomes.

### Implications

Our findings indicate the likely mental health detriment in the years following IPVA exposure in a UK young adult population. The higher levels of depressive symptoms experienced by both women and men who experience IPVA victimisation are also likely to be associated with other adverse health and social outcomes [51–55]. However, our findings suggest that, on average, IPVA may not be a direct cause of the higher burden of depressive symptoms in people who experience IPVA, thus highlighting that IPVA is one of a series of challenges being managed by psychologically vulnerable young people. In addition to supporting young adults who experience IPVA, with strategies for recognising and ending unhealthy relationships, these young adults would likely benefit from support in other areas of their life and life history. Our results were similar in subgroups of women experiencing multiple forms of IPVA, and in women who had also experienced child maltreatment. There was, however, some suggestion of an effect of IPVA on increasing depressive symptoms in men who experienced child maltreatment. In these epidemiological analyses, we are estimating an average effect across a population. These results do not negate the possibility of a causal effect of IPVA on increasing depressive symptoms in some individuals, or in subgroups of the population that we have not examined in these analyses.

Our results also have implications for further research. We adjusted models in two stages and found that estimates only marginally altered when we adjusted for variables beyond those typically adjusted for in previous literature: prior depression scores, ethnicity, socioeconomic status, childhood emotional abuse, physical abuse, sexual abuse, and emotional neglect [6, 7]. However, DiD analyses account for *unmeasured* confounders, and here even evidence of small effects of IPVA on depression scores disappeared. We recommend that a DiD analysis is applied to other longitudinal IPVA and mental health data to investigate whether our findings can be replicated in other populations, as there may be other risk factors for IPVA and depression or effect modifiers that are not being measured in relevant cohorts [5, 56]. Future exploration of such factors, including through qualitative interviews to capture processes not often or easily measured in quantitative studies, could strengthen our understanding of risk factors for experiencing IPVA.

When we examined the relationship between IPVA and depression for different victimisation categories, confidence intervals for the effect of sexual victimisation were wide. However, the point estimate was large (36%; *95% CI* −14 to 113%) for men but not for women (9%, *95% CI* −6 to 26%). Similarly, our sensitivity analysis suggests that men who experience three forms of IPVA (by definition including sexual abuse) have higher levels of depressive symptoms than men who experience one or two forms of IPVA and that the association between IPVA and depressive symptoms is stronger in men who also experienced child maltreatment. Previous studies have highlighted that the patterns of IPVA experienced by male victims tend to differ according to sexual orientation and that sexual violence is more typically experienced by sexual minorities [57]. A systematic review demonstrated that men who have sex with men who have experienced IPVA are more likely to suffer from depressive symptoms, as well as to engage in substance use, be HIV positive, and engage in unprotected anal sex [58]. The number of men reporting sexual violence in our study was small, and the association is imprecisely estimated; we do not have sufficient data to explore whether the effect we see is driven by men who are in a sexual minority group. It is possible that the greater degree of similarity in the mental health impacts across different categories of IPVA for women than for men in our study is a result of women being far more likely than men to suffer frequent ‘multi-victimisation’ or ‘intimate terrorism’ [19, 59].

## Conclusion

In a UK general population sample, young people experiencing intimate partner violence and abuse are likely to have more depressive symptoms than those not victimised. Addressing these symptoms needs to take into account possible exposure to IPVA, and the mental health needs of young survivors of IPVA need to be addressed in the context of IPVA services. However, the causal origins of this increased susceptibility to depression in this population appear to be principally explained by prior vulnerability that increases both depressive symptoms and the risk of IPVA exposure.

## Supplementary Information

**Additional file 1:.** Boxes S1-S4; Figs. S1-S3; Tables S1-S9. Box S1: IPVA questionnaire; Box S2: Antisocial behaviour questionnaire; Box S3: Inverse Probability Treatment Weighting (IPTW) methods; Box S4: Difference-in-difference (DiD) methods; Fig. S1: Propensity score distributions; Fig. S2: Standardised differences; Table S1: Cohort characteristics; Table S2: Logged outcome vs. not (multivariable regression and IPTW); Table S3: Logged outcome vs. not (DiD); Table S4: Comparison of results from different analysis samples (multivariable regression and IPTW); Table S5: Comparison of results from different analysis samples (DiD); Table S6: Exposure number of IPVA types (multivariable regression and IPTW); Table S7: Exposure number of IPVA types (DiD); Fig. S3: Plot of estimated outcome over time, exposure number of IPVA types (DiD); Table S8: Analyses stratified by child maltreatment (multivariable regression and IPTW, complete data); Table S8: Analyses stratified by child maltreatment (multivariable regression and IPTW, complete data)

## Data Availability

ALSPAC data access is through a system of managed open access. The steps below highlight how to apply for access to ALSPAC data. 1. Please read the ALSPAC access policy, available from http://www.bristol.ac.uk/alspac/researchers/access/, which describes the process of accessing the data and samples in detail and outlines the costs associated with doing so. 2. You may also find it useful to browse our fully searchable research proposals database, which lists all research projects that have been approved since April 2011. 3. Please submit your research proposal for consideration by the ALSPAC Executive Committee. You will receive a response within 10 working days to advise you whether your proposal has been approved. If you have any questions about accessing data, please email alspac-data@bristol.ac.uk. The ALSPAC data management plan describes in detail the policy regarding data sharing, which is through a system of managed open access.

## Abbreviations

ACEs: Adverse childhood experiences
ALSPAC: Avon Longitudinal Study of Parents and Children
*CI*: Confidence interval
DiD: Difference-in-difference
IPTW: Inverse probability treatment weighting
IPVA: Intimate partner violence and abuse
MFQ: Moods and Feelings Questionnaire
